# Bisdemethoxycurcumin exerts pro-apoptotic effects in human pancreatic adenocarcinoma cells through mitochondrial dysfunction and a GRP78-dependent pathway

**DOI:** 10.18632/oncotarget.13272

**Published:** 2016-11-10

**Authors:** Haopeng Yang, Shengjun Fan, Yu An, Xin Wang, Yan Pan, Yilixiati Xiaokaiti, Jianhui Duan, Xin Li, Lu Tie, Min Ye, Xuejun Li

**Affiliations:** ^1^ State Key Laboratory of Natural and Biomimetic Drugs, Department of Pharmacology, School of Basic Medical Sciences, Peking University, Beijing 100191, China; ^2^ Beijing Key Laboratory of Tumor Systems Biology, Peking University, Beijing 100191, China; ^3^ Department of Natural Medicines, School of Pharmaceutical Sciences, Peking University, Beijing 100191, China

**Keywords:** bisdemethoxycurcumin, gemcitabine, pancreatic cancer, apoptosis, proteomics

## Abstract

Pancreatic cancer is a highly aggressive malignancy, which is intrinsically resistant to current chemotherapies. Herein, we investigate whether bisdemethoxycurcumin (BDMC), a derivative of curcumin, potentiates gemcitabine in human pancreatic cancer cells. The result suggests that BDMC sensitizes gemcitabine by inducing mitochondrial dysfunctions and apoptosis in PANC-1 and MiaPaCa-2 pancreatic cancer cells. Utilizing two-dimensional gel electrophoresis and mass spectrometry, we identify 13 essential proteins with significantly altered expressions in response to gemcitabine alone or combined with BDMC. Protein-protein interaction network analysis pinpoints glucose-regulated protein 78 (GRP78) as the key hub activated by BDMC. We then reveal that BDMC upregulates GRP78 and facilitates apoptosis through eIF2α/CHOP pathway. Moreover, DJ-1 and prohibitin, two identified markers of chemoresistance, are increased by gemcitabine in PANC-1 cells. This could be meaningfully reversed by BDMC, suggesting that BDMC partially offsets the chemoresistance induced by gemcitabine. In summary, these findings show that BDMC promotes apoptosis through a GRP78-dependent pathway and mitochondrial dysfunctions, and potentiates the antitumor effect of gemcitabine in human pancreatic cancer cells.

## INTRODUCTION

Pancreatic cancer is the fourth leading cause of cancer-related death in United States. The average survival from diagnosis to death is only 4-6 months, and overall 5-year patient survival rate remains less than 6% [1]. The poor prognosis in pancreatic cancer is due to the reduced response of patients to chemotherapy and/or radiotherapy. Gemcitabine and erlotinib, the U.S. Food and Drug Administration-approved therapies against pancreatic cancer, produce objective responses in <10% of the patients [2]. Though it extends survival by mere 3-6 weeks, gemcitabine (GEM) has been recognized as the first-line single treatment against pancreatic cancer for decades. On top of that, GEM has been proposed in combination regimens to treat non-small cell lung carcinoma (with cisplatin), ovarian cancer (with carboplatin), bladder cancer (with cisplatin) and breast cancer (with paclitaxel) [3]. Though many attempts aimed at sensitizing gemcitabine have been evaluated in recent years, investigations into the specific mechanisms of drug combinations encounter many difficulties including the lack of research strategies and the multi-target traits. Thus, optimized analytical methods are urgently needed to investigate detailed mechanisms and to deliver potent treatments against pancreatic cancer.

Curcumin (diferuloylmethane) is a major constituent of the yellow spice turmeric derived from the rhizomes of *Curcuma longa* [4]. It has been well documented that curcumin is a safe and nontoxic agent with demonstrable anti-inflammatory, antioxidant, and antitumor properties [5, 6]. So far, curcumin is one of the most effective agents to improve the current antitumor drugs in clinic. However, due to the limited pharmacokinetic profile of curcumin, intensive studies have shifted to the development of curcumin analogues. Accumulating evidence suggests that curcumin analogues with improved potency and antineoplastic activities be the better therapies for certain types of cancers [7]. Among these curcuminoids, BDMC and desmethoxycurcumin (DMC) are more stable in physiological conditions than the lead compound is [8]. To date, BDMC and DMC have not been investigated whether they exhibit antitumor effects to the same extent as curcumin does. Moreover, mechanisms underlying the antitumor properties of these natural products need to be elucidated to develop effective combination regimens against human cancers.

In the present study, proteomics assays combined with computational bioinformatics are adopted to investigate the specific mechanisms by which BDMC efficiently inhibits the viability of chemoresistant pancreatic cancer cells. As reported that PANC-1 cells display the most resistance to gemcitabine [9], two-dimensional electrophoresis (2-DE) and mass spectrometry (MS) are performed in PANC-1 cells treated with GEM alone or combined with BDMC to disclose the protein expression profiles. Utilizing protein-protein interaction database, GRP78 is identified as the key hub stimulated by BDMC, and the correlated interaction clusters are herein investigated. Together, the results demonstrate that BDMC causes mitochondrial dysfunction and induces apoptosis in human pancreatic cancer cells at a concentration that is significantly lower than that of curcumin. Also, our study reveals that BDMC promotes apoptosis via a GRP78-dependent pathway and counteracts GEM-induced chemoresistance. Thus, we propose BDMC as a promising treatment for human pancreatic cancer.

## RESULTS

### BDMC augments the antitumor effects of GEM in human pancreatic cancer cells

**Figure 1:**
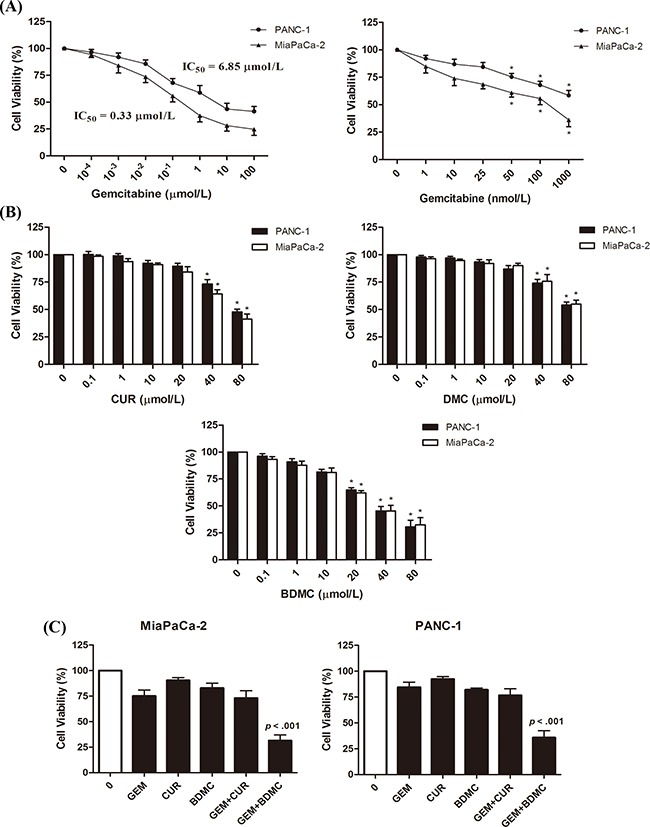
Inhibitory effects of GEM, CUR, DMC, and BDMC in human pancreatic cancer cell **A.** cells were treated with GEM of indicated concentrations for 24h in 96-well plates. **B.** cells were treated with CUR, DMC, and BDMC of indicated concentrations for 24h. Drug efficacy was determined by detecting cell viabilities. **C.** MiaPaCa-2 and PANC-1 cells were treated with GEM (25nmol/L), BDMC (10μmol/L), or the combination for 24h. CUR was used as a comparative drug. Bars are means ± SD from four independent experiments, * *p* < 0.01.

We first evaluated the IC_50_ of GEM in PANC-1 and MiaPaCa-2 cells (Figure 1(A), left), and examined the dose-effect curve from 1nmol/L to 1μmol/L in both cell lines (Figure 1(A), right). We determined 25nmol/L, a dose of no significance, as the concentration of GEM in the following combination treatments. By comparing the effects of curcuminoids on cell viability, we found that BDMC exhibited the highest efficacy in augmenting the inhibitory effects of GEM in MiaPaCa-2 cells (Figure 1(C), left) and PANC-1 cells (Figure 1(C), right). In specific, according to dose-effect curves (Figure 1(B)), BDMC shows significance at 20μmol/L and reduces the viability by nearly 40%. However, curcumin (CUR) or DMC shows little impact at the same concentration. Regarding combination regimen, we determined 10μmol/L, a dose of no significance, as the concentration of BDMC in combination treatments. As shown in Figure 1(C), we compared BDMC with CUR, and found that BDMC-GEM is meaningfully more advantageous than CUR-GEM, which simply shows an additive effect of CUR and GEM. However, BDMC-GEM combination reduces MiaPaCa-2 and PANC-1 cell viability by 68% and 63% respectively, and noticeably shows a greater efficacy than the aggregate of BDMC and GEM does, suggesting a synergy between BDMC and GEM. These results suggest that BDMC is significantly superior to CUR in reducing the viability of pancreatic cancer cell. Moreover, as shown in Table 1, the addition of BDMC decreased the IC_50_ of GEM from 6.85μmol/L to 79.44nmol/L in PANC-1 cells and from 0.33μmol/L to 37.18nmol/L in MiaPaCa-2 cells. Together, these data demonstrate that BDMC alone reduces the viability of MiaPaCa-2 and PANC-1 cell, and improves the efficacy of GEM efficiently in comparisons with curcumin and DMC.

**Table 1:** Changed IC_50_ of gemcitabine in PANC-1 and MiaPaCa-2 cells with BDMC administration

Gemcitabine IC_50_	PANC-1	MiaPaCa-2
0 μM BDMC	6.85 μM	0.33 μM
10 μM BDMC	79.44 nM**	37.18 nM**

** *p* < 0.01 (Paired t test, two tailed)

### BDMC potentiates GEM in pancreatic cancer cells by inducing apoptosis

**Figure 2:**
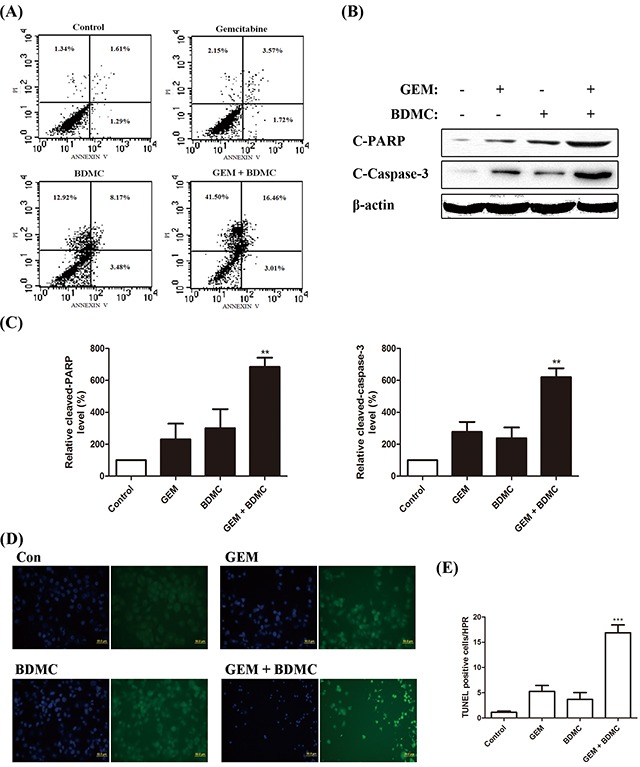
BDMC potentiates GEM in pancreatic cancer cells by inducing apoptosis **A.** cells were treated with GEM (25nmol/L), BDMC (10μmol/L), or the combination for 24h. Cells were analyzed for apoptosis by flow cytometry. **B.** cleaved-PARP and cleaved-Caspase-3 were detected by western blot. **C.** densitometric analysis was used for quantification. Values were normalized to β-actin and were compared with Control levels. Data was shown as means ± SD, n = 3, ** *p* < 0.01. **D.** cells treated with indicated drugs were subjected to TUNEL and Hoechst staining reagents. **E.** the number of TUNEL positive cells per HPR was determined, *** *p* < 0.001.

To evaluate whether the decrease in cell viability induced by BDMC was apoptotic, we adopted flow cytometry to detect the presence of cell death, and found apoptosis occurred in more than 60% of the cells treated with BDMC-GEM combination (Figure 2(A)). We also explored the contents of cleaved-PARP and cleaved-caspase-3 by western blot (Figure 2(B)). Significantly, an increase in the activity of PARP (Figure 2(C), left) and caspase-3 (Figure 2(C), right) was confirmed respectively. Using TUNEL assay and Hoechst staining, cells treated with BDMC-GEM showed early apoptosis (Figure 2(D)). The number of TUNEL-positive cells per HPR was significantly increased in the combination group (Figure 2(E)). These results suggest that BDMC enhances the antitumor effect of GEM by inducing apoptosis.

### BDMC induces apoptosis through mitochondrial dysfunctions

**Figure 3:**
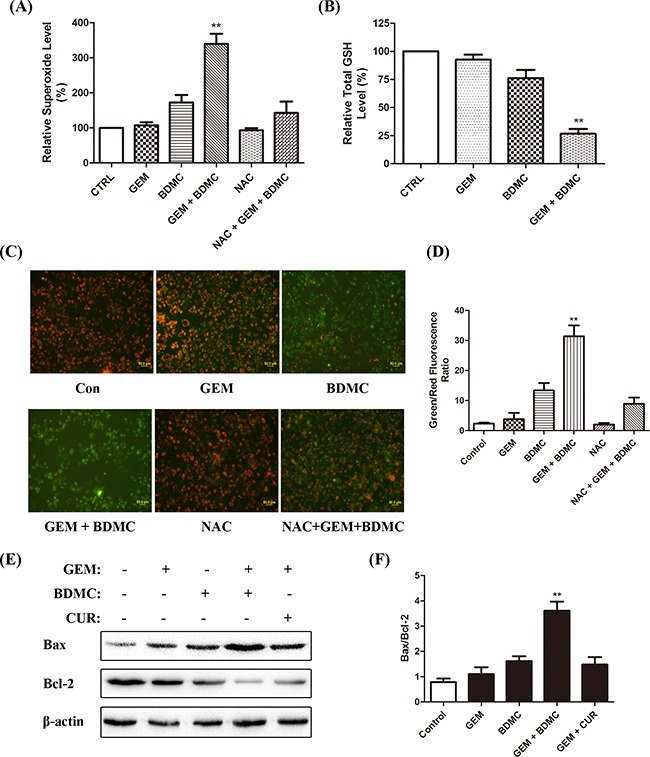
BDMC induces apoptosis through mitochondrial dysfunctions **A.** cells were placed together with WST-1 working solution for 3min at 37°C. Intracellular superoxide levels were determined. **B.** cells were treated with indicated drugs for 24h. Cell lysates were prepared and reacted with assay solutions for 5min at 25°C. Total GSH was determined by standard curves. **C.** representative cell images of JC-1 staining. NAC was pre-incubated for 1h at 200μmol/L. **D.** fluorescent intensity of red and green channels was quantified. Δψm was calculated as red to green fluorescence ratio. **E.** Bax and Bcl-2 were detected by western blot. Curcumin was utilized as a comparative drug. **F.** densitometric study was utilized to quantify protein levels. Values were normalized to β-actin, and data was shown as means ± SD, n = 3, ** *p* < 0.001.

Abnormal oxidative-stress response and the imbalanced levels of intracellular superoxide anions and glutathione (GSH) are both closely associated with CUR-induced apoptosis in various types of cancer [10–12]. We investigated the effect of BDMC-GEM on superoxide production and total GSH levels in PANC-1 cells, and observed an increase in the intracellular superoxide production (Figure 3(A)) and a decrease in total GSH (Figure 3(B)). To evaluate mitochondrial function, we utilized fluorescent JC-1 probe, which forms aggregates in intact mitochondria and emits red fluorescence, while forms monomers upon depolarization and emits green fluorescence [13]. BDMC-GEM treatment caused a significant reduction in mitochondrial membrane potential (Δψ) in PANC-1 cells (Figure 3(C, D)). Also, a significant increase in the ratio of Bax to Bcl-2 was detected in cells treated with the combination (Figure 3(E, F)). Curcumin was adopted as a comparative drug, which consistently showed less efficacy in comparison with BDMC. These results demonstrate that BDMC brings about mitochondrial dysfunctions in pancreatic cancer cells.

### NAC partially reverses BDMC-induced apoptosis in human pancreatic cancer cells

**Figure 4:**
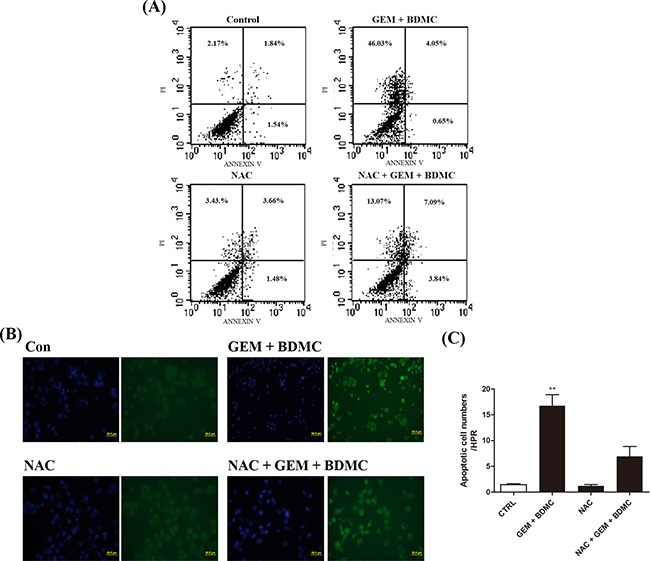
NAC partially reverses BDMC-induced cell death Cells were incubated in 6-well plates and were treated with BDMC-GEM combination for 24h. NAC was pre-incubated before relevant staining reagents were added. **A.** cells were detected for apoptosis using PI and Annexin-V. **B.** representative images of TUNEL staining for detection of cell death. **C.** TUNEL positive cells were quantified as cell numbers per HPR. Values were shown as mean ± SD from three independent experiments, ** *p* < 0.001.

Antioxidant N-Acetyl Cysteine (NAC) was utilized to rescue BDMC-induced apoptosis. Flow cytometry results showed that the pre-incubation of 200 μM NAC for 1 h could partially reverse the apoptotic events by 25% in cells treated with BDMC-GEM (Figure 4(A)). TUNEL assay presented similar results after the pretreatment with NAC (Figure 4(B, C)). These data verified the involvement of oxidative stress in the apoptotic process triggered by BDMC.

### 2-DE and MS assays identify iconic signaling proteins underlying the BDMC-induced apoptosis

**Figure 5:**
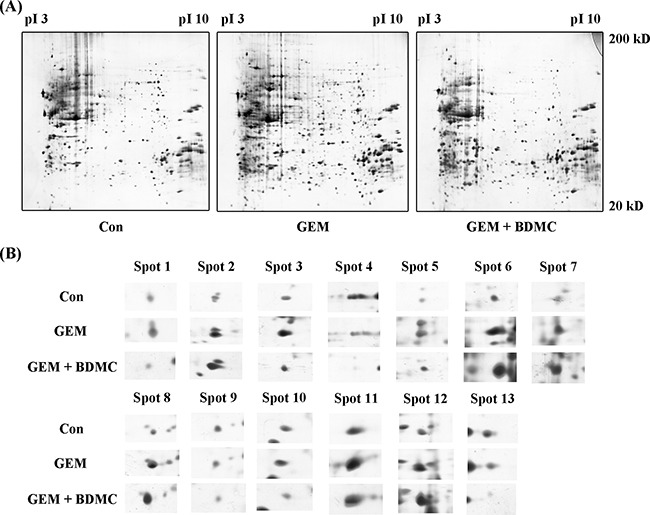
Representative 2-DE gel images **A.** representative 2-DE gel images of control, GEM, and BDMC-GEM groups were listed and stained with silver staining solution. **B.** expanded spots of differentially expressed proteins were identified by MS.

Representative 2-DE gel images for Control, GEM, and BDMC-GEM groups are shown in Figure 5(A). PDQUEST software was adopted to compare the protein maps. Significantly altered spots were identified as pivotal signaling proteins, and were used to establish a whole-protein interaction network underlying the molecular mechanism of BDMC-GEM combination. In total, 13 differentially expressed proteins were revealed and shown in Figure 5(B). Detailed information of these signaling proteins was collected and summarized in Table 2.

**Table 2:** List of differentially expressed proteins identified by mass spectrometry

Spot	Target Protein Name	UniProtKB No.	Gene Symbol	Match	Score
1	Protein DJ-1	Q99497	PARK7	27/29	646
2	Stress-70 protein, GRP 75	P38646	HSPA9	13/17	543
3	Lamin-A/C	P02545	LMNA	73/96	2508
4	Fibroblast growth factor receptor 3	P22607	FGFR3	2/2	150
5	Heterogeneous nuclear ribonucleoprotein	P14866	HNRPL	7/16	123
6	Heterogeneous nuclear ribonucleoprotein D-like	014979	HNRDL	5/6	98
7	Mitochondrial import inner membrane translocase subunit TIM50	Q3ZCQ8	TIM50	11/14	249
8	78kD glucose-regulated protein, GRP78	P11021	HSPA5	35/61	1018
9	Prohibitin	P35232	PHB	13/18	283
10	Annexin A2	P07355	ANXA2	18/24	462
11	T-complex protein 1 subunit beta	P78371	CCT2	12/17	208
12	Pyruvate kinase isozymes	P14618	PKM	23/30	651
13	Malate dehydrogenase, mitochondria	P40926	MDHM	95/110	2888

### Protein-protein interaction network analysis reveals the hub protein

**Figure 6:**
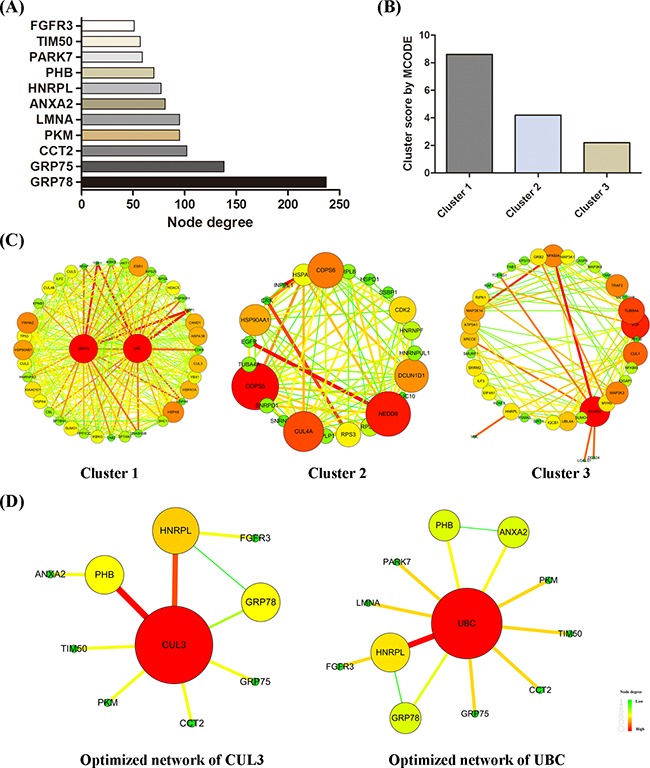
Protein-protein interaction network construction A whole-protein interaction network was established. **A.** node degree of each signaling protein was calculated. **B.** MCODE scores of three subclusters were determined by MCODE algorithm. Subcluster-1 presented the highest score. **C.** three subclusters were shown here. **D.** optimized minimum networks of CUL3 and UBC were respectively created.

Integrated with six protein-protein interaction databases, 13 signaling proteins identified by MS assay were input into Cytoscape working station to construct a whole-protein network, which was further analyzed for subclusters via BisoGenet. MCODE algorithmic calculations and Prioritizer analysis were performed to evaluate the subclusters and to predict pivotal hubs in the network. By calculating node degree, GRP78 was identified as the most important node with the highest degree in the network (Figure 6(A)). MCODE algorithm recognized three major subclusters, among which subcluster 1 showed the highest score (Figure 6(B, C)).

Via Prioritizer plugin, the indicated network was screened in parallel for six parameters, namely Generalized Random Walk Receiver Closeness, Generalized Shortest Path Closeness, Generalized Random Walk Betweenness, Generalized Degree Centrality, Generalized Random Walk Transmitter Closeness, and Generalized Shortest Path Betweenness. The ranking of hub proteins under all parameters were accordingly created and shown in Table 3. CUL3 and UBC were two genes occurred across all top-five rankings. By integrating with MS results, two optimized minimum networks converged with CUL3 (Figure 6(D), left) and UBC (Figure 6(D), right) were constructed respectively. Such optimized networks are purely composed of protein nodes that link to each other by single edge or by only one intermediate partner.

**Table 3:** Rankings of possible hub proteins identified by BisoGenet

Rank	Generalized Random Walk Receiver Closeness	Generalized Shortest Path Closeness	Generalized Random Walk Betweenness	Generalized Degree Centrality	Generalized Random Walk Transmitter Closeness	Generalized Shortest Path Betweenness
1	UBC*	UBC	UBC	UBC	UBC	UBC
2	SPTAN1	CUL3	GRB2	HSPA8	CUL3	SUMO2
3	CUL3*	FN1	CUL3	CUL3	SUMO2	HSP90AA1
4	SUMO2	SUMO1	APP	ESR1	SPTAN1	CUL3
5	ESR1	YWHAZ	HSP90AB1	HSP90AA1	ESR1	HSP90AB1

* Gene symbols that occurred across all six rankings

### BDMC upregulates GRP78 and triggers cell transition from ER stress to apoptosis through eIF2α/CHOP pathway

**Figure 7:**
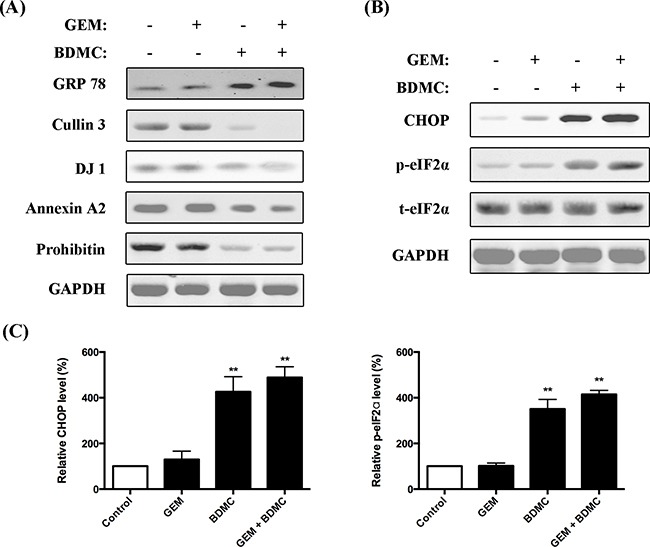
Evaluations on protein interaction network **A.** immunoblotting analysis on GRP78, Cullin 3, DJ-1, Annexin A2, Prohibitin and GAPDH in cells treated with GEM, BDMC, and the combination. **B.** expression levels of CHOP, p-eIF2α and t-eIF2α were determined. **C.** densitometric analysis was used to quantify the levels of CHOP and p-eIF2α. Values were normalized to GAPDH. Data was shown as mean ± SD from three independent experiments, ** *p* < 0.001.

To examine the results proposed by the bioinformatics analysis, we investigated the signaling proteins by western blot assays. In consistency with 2-DE data, we observed a significant upregulation of GRP78 in cells treated with BDMC alone or together with GEM (Figure 7(A)). Cullin3, the other key node indicated by the network analysis, is meaningfully downregulated by BDMC in PANC-1 cells. Eukaryotic translation initiation factor alpha (eIF2α) constitutes the essential pathway underlying the unfolded protein response (UPR) [14, 15]. We found that the phosphorylation of eIF2α was significantly increased by BDMC (Figure 7(B, C-right)). Furthermore, we observed an enhanced expression of C/EBP homologous protein (CHOP) in cells treated with BDMC alone or together with GEM (Figure 7(B, C-left)). Together, these results suggest that BDMC upregulates GRP78 and transfers cells from ER stress to apoptosis through eIF2α/CHOP pathway.

### BDMC offsets GEM-induced chemoresistance in pancreatic cancer cells

As suggested in the network construction, the optimized minimum networks pinpoint three chemoresistance markers, namely protein DJ-1, annexin-A2, and prohibitin, which have shown close relationship to chemoresistance in various types of cancer [16–18]. As demonstrated in Figure 7(A), DJ-1, annexin-A2, and prohibitin were significantly downregulated by BDMC alone or combined with GEM. This was consistently detected by the MS assays shown in Figure 5. These data indicate that BDMC counteracts the chemoresistance induced by gemcitabine in pancreatic cancer cell.

### A GRP78-dependent pathway was revealed

**Figure 8:**
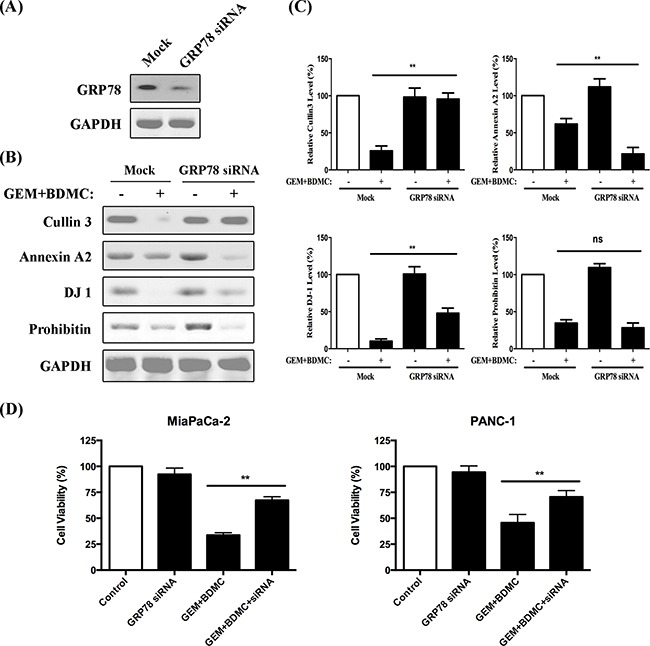
A GRP78-dependent pathway was revealed **A.** GRP78 expression was evaluated after siRNA administration. **B.** western blots of Cullin3, Annexin A2, DJ-1, and Prohibitin were presented. **C.** densitometric analysis was utilized to quantify the protein content. Values were normalized to GAPDH and were compared with control group; ns, no significant difference. **D.** cell viability was determined in MiaPaCa-2 and PANC-1 cells, respectively. Data was shown as mean ± SD from three independent experiments, ** *p* < 0.01.

Expression of GRP78 was shown in Figure 8(A) after the transient transfection of siRNA. To investigate whether the downregulations of Cullin3, Annexin-A2, DJ-1 and Prohibitin were dependent on GRP78, we compared their expressions in BDMC-GEM-treated cells with and without GRP78 siRNA. Cullin3, annexin-A2, and DJ-1 showed significant changes in expression levels, and were thereby revealed as the downstream molecules of GRP78 (Figure 8(B)). However, the inhibition of prohibitin was independent on GRP78 (Figure 8(C)).

### GRP78 siRNA partially reverses BDMC-GEM-induced apoptosis in pancreatic cancer cells

For rescue experiments, we examined the viability of MiaPaCa-2 and PANC-1 cells transfected with GRP78 siRNA. The results showed that GRP78 siRNA partially reversed the BDMC-GEM-induced apoptosis by approximately 35% in MiaPaCa-2 cells (Figure 8(D), left) and 25% in PANC-1 cells (Figure 8(D), right).

**Figure 9:**
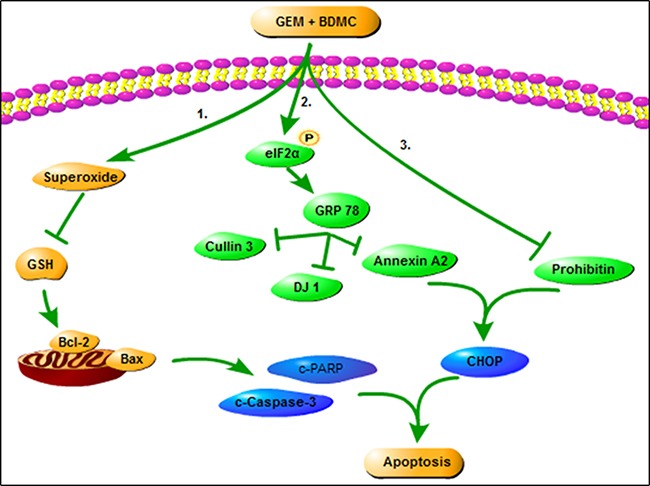
The role of BDMC in sensitizing GEM On one hand, BDMC promotes cell death by inducing mitochondrial dysfunction, activates GRP78, and facilitates cell transition from ER stress to apoptosis. On the other hand, BDMC inhibits DJ-1 and prohibitin, and dispels GEM-induced chemoresistance in pancreatic cancer cell.

Taken together, our results reveal that BDMC induces mitochondrial dysfunctions in pancreatic cancer cells, and facilitates apoptosis through a GRP78-dependent pathway (Figure 9). Thus, we propose BDMC for further clinical studies to treat pancreatic cancer.

## DISCUSSION

Here, we have shown that BDMC, a derivative of curcumin, is significantly more effective in promoting apoptosis in gemcitabine-resistant pancreatic cancer cells in comparisons with other curcuminoids, and we elucidate its specific mechanisms. Initially, this could be due to the induction of mitochondrial dysfunctions and abnormal oxidative-stress response in cells treated with BDMC. Oxidative stress refers to an intracellular situation of an imbalance between pro-oxidant and antioxidant factors. It causes cellular damage that is primarily generated from mitochondria [10]. Mitochondrial defects lead to imbalanced redox environments within cells, eliciting abnormal oxidative-stress response that either activates severe mitochondrial pathogenesis or triggers DNA damage [19], cellular damage [20], and/or other apoptotic events [21]. Curcumin, a natural chemopreventive agent that is nontoxic but highly effective in killing cancer cells, has shown multi-targeted characteristics [22, 23], including disrupting redox balance in tumor cells [24]. In line with this, our results elucidate that BDMC causes an increased level of intracellular superoxide anions as well as a reduction in total GSH content in cells treated with either BDMC alone or combined with gemcitabine. Also, we observe that BDMC brings about a significant reduction in mitochondrial membrane potential and a meaningfully increased ratio of Bax to Bcl-2. Bax and Bcl-2 partially locate on mitochondrial membrane and are closely related to cell death and survival. These data indicate that mitochondrial dysfunctions and an imbalanced redox condition are induced in the cells treated with BDMC. The hypothesis that BDMC inhibits PANC-1 cell viability through redox stress is also evidenced by the rescue experiments utilizing NAC pre-treatment. These results thereby suggest that BDMC induces cell death via mitochondrial dysfunctions and oxidative stress in human pancreatic cancer cells.

Pathological stimuli such as DNA damage and oxidative stress impede endoplasmic reticulum (ER) functions, resulting in the accumulation of unfolded proteins and then ER stress in cells. It is known that oxidative stress and ER stress are interlinked via multiple pathways including redox imbalance and reactive oxygen species (ROS) overproduction [25, 26]. GRP78, an ER-located chaperon, mediates essential components of ER stress-induced apoptosis, and is considered as an ER-stress marker [27, 28]. Significantly upregulated GRP78 indicates the inductions of Unfolded Protein Response (UPR) and ER stress [29]. Here, our results show for the first time that BDMC upregulates GRP78 in GEM-resistant PANC-1 cells, which consistently has been elucidated by MS and the following immunoblotting assays. Furthermore, cancer cells progressing towards UPR and ER stress provide either survival or cell death signals, which primarily depends on the extent of ER stress occurred in cells [14, 30]. Cells exposed to moderate ER stress can avoid apoptosis by undergoing UPR or by activating autophagy [31], while cells exposed to severe and protracted ER stress tend to launch programmed apoptosis via multiple steps [27], wherein CHOP is a well-characterized marker for the cell transition from ER stress to apoptosis [30, 32]. Recent research also suggests that pharmacological interventions inducing prolonged ER stress may be targeted for cancer therapy [33, 34]. We observe that BDMC meaningfully increases CHOP and activates the phosphorylation of eIF2α. These together suggest that BDMC causes ER stress and eventually triggers the cell transition from ER stress to apoptosis, which may partially correlate with the mitochondrial dysfunction and oxidative stress induced by BDMC.

By integrating MS data with bioinformatics resources, several chemoresistance-related targets are identified and further studied to fully understand the antitumor effects of BDMC. Cullins are a family of proteins that confer substrate specificity to the multimeric complex of E3 ligases acting as scaffold proteins. Interestingly, distinguished from other family members, cullin3 has been implicated to have a special relationship with redox homeostasis and intracellular stress responses [35]. Cullin3 has been targeted to develop therapeutic strategies for redox disorders. Besides, recent research has shown that cullin3 correlates with cancer progression and is evidenced as a promising target to treat human cancers [36, 37]. Notably, cullin3 is significantly overexpressed within cancer sites but remains undetectable in normal tissues [38]. Here, we discover that cullin3 is noticeably downregulated in cells treated with either BDMC alone or together with GEM. Depletion of GRP78 significantly prevents the inhibition of cullin3, suggesting cullin3 inhibition is dependent on the activation of GRP78 by BDMC. In light of the above bioinformatics analysis, GRP78 and cullin3 collocate in the same minimum protein network (Figure 6(D)). Besides, GRP78 is presented with a relatively higher node degree which is indicated in a dark yellow tag in the optimized network of CUL3. Together, these results strongly indicate that potential interplays between GRP78 and cullin3 may occur in the intracellular stress responses induced by BDMC. Of note, the above results highlight that BDMC inhibits cullin3 in pancreatic cancer cells, which is dependent on the activation of GRP78.

Additionally, DJ-1 and prohibitin, which have been extensively reported to closely correlate with chemoresistance and to overexpress within cancer sites [16–18], are both suppressed in cells treated with BDMC alone or combined with GEM. The downregulation of DJ-1 could be significantly reversed by depletion of GRP78. However, the difference of prohibitin content between siRNA +/− groups is not meaningful, indicating that the inhibition of prohibitin by BDMC is GRP78-independent. Annexin A2 is an inducible, calcium-dependent phospholipid-binding protein, and is also overexpressed in a variety of human malignancies [39, 40]. Recent studies have shown that curcumin potentially inhibits Annexin A2 in cancer tissues [41]. Here, our results show that Annexin A2 is differentially expressed in response to GEM alone and BDMC-GEM combination. We demonstrate that BDMC represses Annexin A2 in pancreatic cancer cells, which is also dependent on GRP78 activation. Taken together, these results elucidate that BDMC offsets the chemoresistance induced by gemcitabine in human pancreatic cancer cells.

To summarize, compared with curcumin and DMC, BDMC possesses the greatest efficacy in sensitizing gemcitabine in pancreatic cancer cells. On one hand, BDMC causes mitochondrial dysfunctions and triggers a GRP78-dependent pathway to directly promote apoptosis; on the other hand, it eliminates the chemoresistance induced by gemcitabine. Herein, we propose BDMC-GEM combination regimen as a better solution to treat pancreatic cancer.

## MATERIALS AND METHODS

### Reagents and cell culture

Gemcitabine, Curcumin, DMC and BDMC were from Sigma-Aldrich Corporation (St. Louis, MO, USA). Anti Bcl-2, Bax, Activated-Caspase-3, Activated-PARP, GRP78 and CHOP antibodies were from Bioworld Technology, Inc. (Louis Park, MN, USA). Antibody to Cullin3 was from US Bio (MA, USA), and antibodies to Annexin A2, Prohibitin, p-eIF2α and eIF2α were obtained from Cell Signaling Technology, Inc. (Danvers, MA, USA). Unless specifically indicated, all other biochemical reagents were obtained from Amresco (Solon, OH, USA). PANC-1 and MiaPaCa-2 cell lines were from Cell Culture Center, Institute of Basic Medical Science, Chinese Academy of Medical Sciences (Beijing, China). Briefly, PANC-1 and MiaPaCa-2 cells were cultured in DMEM medium supplemented with 10% fetal bovine serum (FBS; Hyclone, Logan, Utah, USA) in a humid atmosphere incubator with 5% CO_2_ at 37°C. Cells were treated with drugs or vehicles after growing to 70% to 80% confluence.

### Cell viability assay

Cell viability was measured by a commercial MTS assay kit (Promega, Fitchburg, Wisconsin, USA). Cells were seeded into 96-well plates at a density of 4,000 cells/well. Drug treatments of indicated concentrations were added for 24 hours. The absorbance was determined at 490nm by a micro-plate reader (Thermo Scientific, Menlo Park, CA, USA) 4 hours after the probe reagents had been added.

### Flow cytometric analysis

Cells were diluted to 8 × 10^4^/ml and incubated for 48h at 37°C in 6-well plates. Cells were then treated with control solvent (0.1% DMSO), GEM (25nmol/L), BDMC (10μmol/L) alone or their combination for 24 hours. Cells were collected, washed in PBS, and were resuspended in 100μl binding buffer containing 5μl of Annexin V-FITC (Pharmingen, San Diego, CA) and 5μl of PI (Pharmingen, San Diego, CA). Samples were mixed gently and incubated at room temperature in dark for 20min. 500μl binding buffer was then added to each sample tube. Samples were analyzed by FACS (Beckman Coulter, CA). A minimum of 10,000 cells within gated region were collected.

### TUNEL staining

Apoptosis was measured using *in situ* cell-death detection kit (G3250, Promega, Madison, USA) as we previously described [42]. Terminal deoxynucleotidyl transferase biotin-dUTP nick end labeling (TUNEL) staining was performed according to the manufacturer's instructions. TUNEL-positive cells were indicated in green fluorescence in Figures 2(D) and 4(B).

### Measurement of intracellular superoxide and total GSH levels

Cells were seeded into 6-well plates, and were incubated for 24 hours. Commercial intracellular superoxide and t-GSH kits (Beyotime, Beijing, China) were utilized to determine the levels of intracellular SOD and t-GSH. According to manufacturer's brochure, the values were measured by a micro-plate reader (Thermo Scientific, Menlo Park, CA, USA) for quantifications.

### Determination of mitochondrial membrane potential (Δψm)

JC-1 fluorescent probe kit (Beyotime, Beijing, China) was used to determine Δψm. Briefly, cells were incubated in DMEM media containing JC-1 (5μg/ml) for 30min at 37°C, and were then measured by fluorescent microscope (Carl Zeiss, Axiovert 200). Δψm was determined by MPF-66 fluorescence spectrometer (Perkin-Elmer) with excitation wavelength at 490nm and emission wavelength at 530/590nm. The ratios of readings at according wavelengths were converted into relative Δψm values.

### Two-dimensional gel electrophoresis and mass spectrometry identification

Cells were randomly divided into three groups: control, GEM (25nmol/L, 24 h), and BDMC-GEM (10μmol/L BDMC, 25nmol/L GEM, 24 h). 2-DE and MS assays were performed as we previously described [43]. Cells were collected, washed with PBS, and subsequently centrifuged for 1min at 2,500g. Cell pellets were dissolved in RIPA lysis buffer, and were ultra-sonicated (ten strokes, low amplitude) on ice for homogenization. Lysed cells were centrifuged at 15,000g for 30min at 4°C. Supernatant containing solubilized proteins was used for 2-DE and MS experiments. Bio-Rad 2-DE system was utilized, and 200μg protein sample was applied for IEF using ReadyStrip IPG Strips (17cm, pH 4-7) (Bio-Rad). The strips were placed into Protein IEF (Bio-Rad), and were rehydrated at 50V for 12h. Proteins were thus separated by *pI* values. IPG strips were then equilibrated and directly applied for 12% homogeneous SDS-PAGE gels electrophoresis using PROTEIN II xi Cell System (Bio-Rad). Gels were stained using Silver Stain Plus Kit reagents (Bio-Rad). Protein samples were obtained from three independent experiments. Stained gels were scanned by Densitometer GS-800 (Bio-Rad) and were analyzed via PD-Quest software (Bio-Rad). Protein spots were excised from gels with EXQuest Spot Cutter (Bio-Rad) for following MS identifications using ABI 4700 Proteomics Analyzer (Framingham, MA, USA).

### Protein-protein interaction network construction

Protein-protein interaction information from six publicly available databases including Human Protein Reference Database [44], Molecular Interaction database [45], IntAct [46], Database of Interacting Proteins [47], Biogrid [48], and MIPS [49] was integrated as we previously described [50, 51]. Signaling hub proteins indicated by MS studies as well as their protein-interaction database information were integrated and input into BisoGenet and Cytoscape Working Platforms to expand and construct their corresponding whole-protein network. To further nail down specific clusters correlated to BDMC-GEM combination, MCODE algorithm and Prioritizer plugin were adopted. Non-associated intermediate nodes were removed. Optimized minimum networks containing key hubs were thereby constructed.

### Cell transfection and GRP78 RNAi assays

Commercial GRP78 siRNA was obtained from Santa Cruz Biotechnology, Inc. (Santa Cruz, CA, USA). GRP78 siRNA at a concentration of 100 nmol/L was transiently transfected into PANC-1 cells with Lipofectamine-2000 reagent (Invitrogen, Carlsbad, CA, USA). After incubation for 24 hours, PANC-1 cells were harvested for further experiments.

### Immunoblot assays

PANC-1 cells were harvested and lysed in RIPA buffer containing PMSF (phenylmethylsulfonyl fluoride) and protease inhibitor cocktail (Calbiochem, San Diego, CA, USA). Following centrifugation at 12,000g for 15min at 4°C, supernatants were collected. Protein concentration was measured by bicinchoninic acid (BCA) assay kit (Thermo Scientific Pierce, Rockford, IL, USA). Protein samples (80μg) were separated by 12% sodium dodecyl sulfate-polyacrylamide gel (SDS-PAGE). Upon completion of electrophoresis, proteins were transferred to PVDF membrane. Membranes were then incubated with 5% skimmed milk in Tris-buffered saline Tween (TBS-T, 20mmol/L Tris, 137mmol/L NaCl, pH 7.6) for overnight at 4°C with primary antibodies. After washed three times with TBS-T buffer, membranes were incubated with Alexa Fluor secondary antibodies (Cell Signaling Technology, Inc., Danvers, MA, USA), and were then scanned by Odyssey Image System (LI-COR Biosciences, Nebraska, USA). Band intensity was analyzed by Bio-Rad Quantity One Software (version 4.4.0; Bio-Rad, Hercules, CA, USA) for quantifications.

### Data and statistical analysis

All values are expressed as means ± SD. For multiple comparisons, statistical analysis was performed using one-way analysis of variance (ANOVA) and Newman-Keuls multiple comparison tests. A value of *p* < 0.05 was considered statistically significant.
